# Astaxanthin-, β-Carotene-, and Resveratrol-Rich Foods Support Resistance Training-Induced Adaptation

**DOI:** 10.3390/antiox10010113

**Published:** 2021-01-14

**Authors:** Aki Kawamura, Wataru Aoi, Ryo Abe, Yukiko Kobayashi, Masashi Kuwahata, Akane Higashi

**Affiliations:** 1 Division of Applied Life Sciences, Graduate School of Life and Environmental Sciences, Kyoto Prefectural University, Kyoto 6068522, Japan; aki.kawamura@nittai.ac.jp (A.K.); 7963.in.kyoto@gmail.com (R.A.); yukicoba@kpu.ac.jp (Y.K.); kuwahata@kpu.ac.jp (M.K.); higashi-akane@kpu.ac.jp (A.H.); 2 Sports Science Research Promotion Center, Nippon Sport Science University, Tokyo 1588508, Japan; 3 Department of Nutrition Management, Wakayama Medical University Hospital, Wakayama 6418510, Japan

**Keywords:** muscle adaptation, resistance training, astaxanthin, β-carotene, resveratrol

## Abstract

Resistance training adaptively increases the muscle strength associated with protein anabolism. Previously, we showed that the combined intake of astaxanthin, β-carotene, and resveratrol can accelerate protein anabolism in the skeletal muscle of mice. The purpose of this study was to investigate the effect of anabolic nutrient-rich foods on muscle adaptation induced by resistance training. Twenty-six healthy men were divided into control and intervention groups. All participants underwent a resistance training program twice a week for 10 weeks. Astaxanthin-, β-carotene-, and resveratrol-rich foods were provided to the intervention group. Body composition, nutrient intake, maximal voluntary contraction of leg extension, oxygen consumption, and serum carbonylated protein level were measured before and after training. The skeletal muscle mass was higher after training than before training in both groups (*p* < 0.05). Maximal voluntary contraction was increased after training in the intervention group (*p* < 0.05), but not significantly increased in the control group. Resting oxygen consumption was higher after training in the intervention group only (*p* < 0.05). As an oxidative stress marker, serum carbonylated protein level tended to be lower immediately after exercise than before exercise in the intervention group only (*p* = 0.056). Intake of astaxanthin-, β-carotene-, and resveratrol-rich foods supported resistance training-induced strength and metabolic adaptations.

## 1. Introduction

Resistance exercise training has been implemented to improve athletic performance and prevent non-communicable diseases and sarcopenia. A major adaptive effect of resistance training is increased muscle strength. Generally, transmission and conduction of nervous impulses are activated mainly during the early period of training, which contributes to the contraction of muscle fibers. With time, continuous resistance training causes the hypertrophy of each muscle fiber, which leads to a further increase in strength. Another major function of skeletal muscle is energy expenditure. Muscles consume energy substrates and produce large amounts of ATP used for muscle contraction; this corresponds to approximately 20% of the basal metabolism of the entire body [1]. Therefore, the metabolic rate increases with muscle hypertrophy [2].

Protein synthesis and catabolism occur continuously in the body. Promotion of protein synthesis and prevention of protein catabolism lead to muscle hypertrophy. Resistance training is well known to be the most efficient way of stimulating protein anabolism, which is mediated by various signaling factors. Furthermore, adequate nutritional management accelerates improvements in muscle strength and mass during resistance training. Generally, sufficient intake of protein is required to supply amino acids as substrates for protein synthesis in skeletal muscle cells [3,4]. A combined intake of carbohydrates and proteins is also effective in increasing protein anabolism by stimulating insulin secretion [5]. Many studies have also demonstrated the positive effects of macronutrient intake on training-induced muscle adaptation [3,4,5]. However, studies that investigate the effects of micronutrients on training adaptation are limited.

Previous studies showed that some micronutrients promote protein synthesis and skeletal muscle metabolism in vivo and in vitro. In humans, the administration of astaxanthin, a carotenoid, has been proven to improve the adaptation of muscle strength during resistance training [6]. In animal and culture cell studies, β-carotene, a carotenoid, and resveratrol have been reported to activate protein synthesis signaling factors, Akt, the mammalian target of rapamycin (mTOR), and p70S6 kinase (p70S6K) in skeletal muscles [7,8,9]. These findings suggest that astaxanthin, β-carotene, and resveratrol might enhance anabolism in skeletal muscles. Recently, we found that these anabolism-enhancing micronutrients, astaxanthin, β-carotene, and resveratrol, promoted muscle hypertrophy with elevated protein synthesis in a mouse hypertrophic model [10]. This anabolic effect was accelerated when taken in combination in small doses rather than in a single large-dose intake. These findings led us to hypothesize that dietary foods containing a small dose of anabolic micronutrients may be useful to facilitate muscle adaptation during resistance training in humans. Here, we report that dietary intake of astaxanthin-, β-carotene-, and resveratrol-rich foods supports resistance training-induced adaptation.

## 2. Materials and Methods

### 2.1. Participants

Twenty-six healthy men (age: 22.3 ± 0.3 years; height: 171.1 ± 1.6 cm) were recruited as participants, which was approved by the ethics committee of Kyoto Prefectural University (No. 2016-112) and the university hospital medical information network clinical trials registry in Japan (UMIN000041217). All participants signed a consent form after receiving an explanation of the study. None of the participants had a current or prior chronic disease or history of smoking. None currently used any medication or supplements. In addition, none of the participants were habituated to regular exercise. The participants were randomly divided into two equal-sized groups, the control and intervention groups (*n* = 13), and body composition was measured using bioelectrical impedance analysis (BIA; lnBody430, Kobe Medi-Care Co., Ltd., Hyogo, Japan).

### 2.2. Experiment Design

This study was designed as a randomized controlled trial. All the participants performed the resistance training program twice a week for 10 weeks. During the training period, salmon flakes, green and yellow vegetable juice, and lingonberry jam, which contain astaxanthin, β-carotene, and resveratrol, respectively, were provided for the intervention group. Body composition, nutrient intake, maximal voluntary contraction (MVC), oxygen consumption, subjective fatigue, and serum carbonylated protein level were measured before and after training. Measurements of muscle strength, body composition, and expiratory gas were performed during the week before the training period and during the final week of the period. Blood samples were taken on the first day of exercise and on the day of exercise in the final week of the training period.

### 2.3. Resistance Exercise Training

The resistance training program consisted of eight types of exercise (chest press, chest fly, leg press, leg extension, leg curl, back extension, seated rowing, and sit-up) using a combined machine (Senoh Co., Ltd., Chiba, Japan) and performed in 3 sets of 10 repetitions with 10 repetition maximum (RM). The training frequency was twice a week, with 2 to 3 d intervals set between training sessions, and the load was gradually increased according to the 10 RM for each individual. The MVC of leg extension was measured in both the left and right legs with a leg-extension strength meter (ST 200R, Meiko Co., Ltd., Osaka, Japan).

### 2.4. Indirect Metabolic Performance

The participants were instructed to refrain from intense physical activity, eating, and drinking, except for water, from 22:00 until breakfast in the morning. They were asked to take breakfast without caffeine and alcohol at least 1.5 h before visiting the laboratory. After sitting for 30 min, oxygen consumption and carbon dioxide production were measured in the supine position using a breath-by-breath respiromonitor system (AE-310s, Minato Medical Sciences Co., Ltd., Osaka, Japan) for 20 min. The respiratory quotient and substrate use were calculated from the levels of oxygen consumption and carbon dioxide production [11].

### 2.5. Serum Carbonylated Protein

Carbonylated protein, an oxidative damage marker, was measured before and after the resistance exercise at the pre- and post-intervention periods. The participants were instructed to refrain from intense physical activity and fast from 22:00 on the eve of blood sample collection. On the day of blood sampling, they took steamed rice (200 g, energy 372 kcal, protein 5.0 g, fat 0.6 g, and carbohydrate 75.6 g) and rested for 1 h. A blood sample was collected from the median cutaneous vein. After a resistance exercise session, blood was collected again. The collected blood was injected in a vacuum blood collection tube and centrifuged at 1900× *g* at 4 °C for 15 min. Serum samples were used to measure carbonylated protein concentrations by using a protein carbonyl enzyme-linked immunosorbent assay (ELISA) kit (BioCell Co., Ltd., Auckland, New Zealand) in accordance with the manufacturer’s instructions.

### 2.6. Subjective Fatigue

The degree of subjective fatigue before exercise (at rest) was measured every 2 to 3 weeks during the intervention period by using the visual analog scale (VAS) method. The VAS was used to examine the level of subjective fatigue. The participants were asked to indicate the degree of subjective fatigue on a 100 mm horizontal line. For the left side (0 mm), they stated “having no fatigue”, whereas for the right side (100 mm), they stated “having max fatigue”.

### 2.7. Nutrient Intake

Dietary assessment was conducted to calculate nutrient intake before and at week 10 of training. All the participants were permitted to eat freely, and their food intakes were recorded for 3 d by using a food diary and camera. Then, a dietitian reviewed their diet to follow up and estimate their nutrient intakes by using an Excel add-in software (Excel Eiyou-kun Ver. 6.0, Kenpakusha Co., Ltd., Hokkaido, Japan). For the intervention group, salmon flakes (1 mg astaxanthin/100 g; Fujiyahonten Co., Ltd., Hokkaido, Japan), vegetable juice (4.5 mg β-carotene/100 mL; KAGOME Co., Ltd., Aichi, Japan), and lingonberry jam (0.24 mg resveratrol/100 g; IKEA Japan Co., Ltd., Chiba, Japan) were provided. The participants in the intervention group were asked to consume more foods containing green and yellow vegetables, salmon, and shrimp, in addition to the foods provided. The target intake amounts of the macronutrients and micronutrients were advised by a dietitian according to their individual body weights. All participants in the intervention group had consumed the provided food. By contrast, the control group was neither encouraged nor restricted in their intake of these three ingredients.

### 2.8. Statistical Analyses

All data are reported as mean ± standard error (SE). Differences between the control and intervention groups were tested using the unpaired *t*-test or two-way analysis of variance (ANOVA). When significant differences were determined with ANOVA, post hoc analyses were conducted with the Bonferroni test. For the parameters with the absence of normality, nonparametric statistical analysis was performed. Cohen’s *d* was calculated to measure the effect size between the control and intervention groups. The statistical analysis was completed using SPSS ver. 25 (IBM Japan Inc., Tokyo, Japan), and *p* values < 0.05 were considered statistically significant.

## 3. Results

### 3.1. Body Composition

The skeletal muscle mass was significantly higher after than before training in both groups (control: *p =* 0.002, intervention: *p* = 0.030; Table 1). We found no significant differences in body weight, body mass index, and body fat between before and after training. The changes in these parameters were not significantly different between the groups.

### 3.2. Muscle Strength

**Figure 1:**
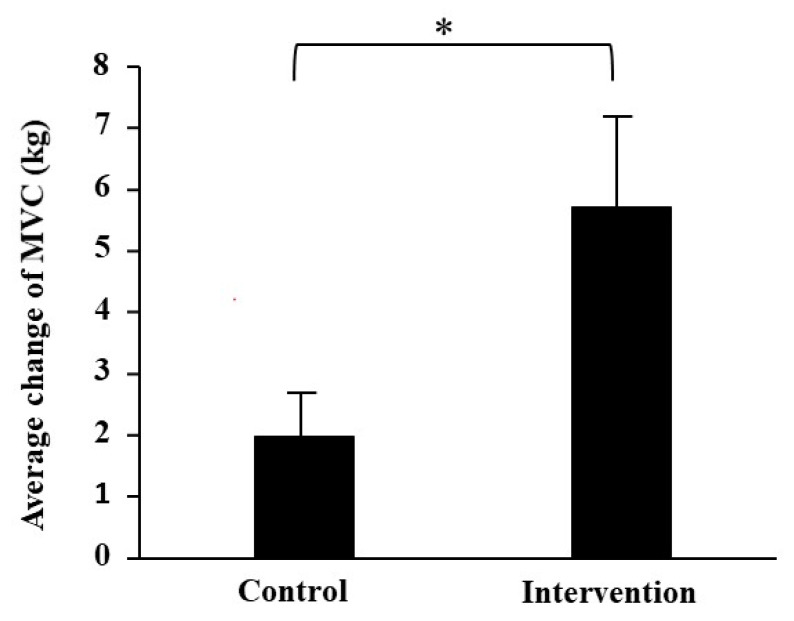
Comparison of the average changes in left and right MVC from before to after resistance exercise training between the two groups. The values are presented as mean ± SE. * Significantly different at *p* < 0.05 compared with the control group.

The MVC of leg extension was significantly increased after training as compared with before training in the intervention group (right: *p* = 0.001, left: *p* = 0.001; Table 1). It showed a tendency to increase after training in the control group (right: *p* = 0.071, left: *p* = 0.071). In addition, the average changes in MVC were significantly higher in the intervention group than in the control group with a large effect size (*p* = 0.040, *d* = −1.474; Figure 1).

### 3.3. Metabolic Performance

In the intervention group, oxygen consumption was significantly higher after than before training (*p* = 0.046, *d* = −0.62), but not in the control group (Table 2). The respiratory quotient was significantly decreased after training as compared with before training for the control group only (*p* = 0.031, *d* = 0.87). In addition, carbohydrate and fat oxidations were significantly changed after training as compared with before training (*p* = 0.049 and *p* = 0.017, respectively).

### 3.4. Serum Carbonylated Protein

**Figure 2:**
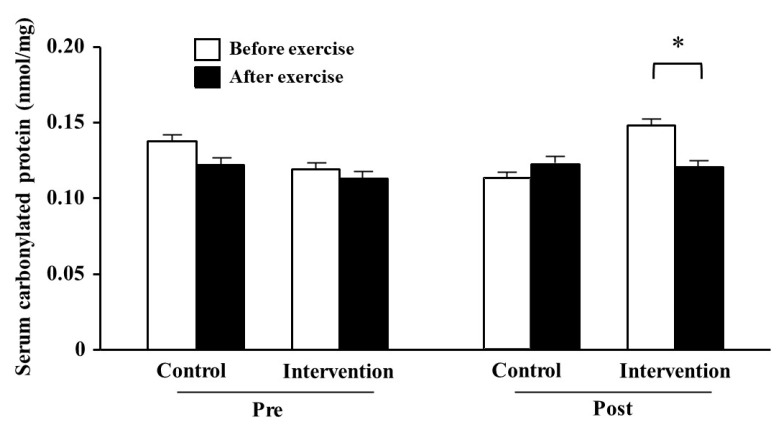
Comparison of serum carbonylated protein level before and after resistance exercise training. The white and black bars indicate the carbonylated protein levels before and after training, respectively. * Difference at *p* = 0.059. The values are presented as mean ± SE. Pre: pre-training, Post: post-training.

The serum carbonylated protein levels before training did not change between before and after exercise in either group (Figure 2). By contrast, it tended to be lower after than before exercise (*p* = 0.059) in the post-training period in the intervention group, but not significantly changed in the control group.

### 3.5. Subjective Fatigue

**Figure 3:**
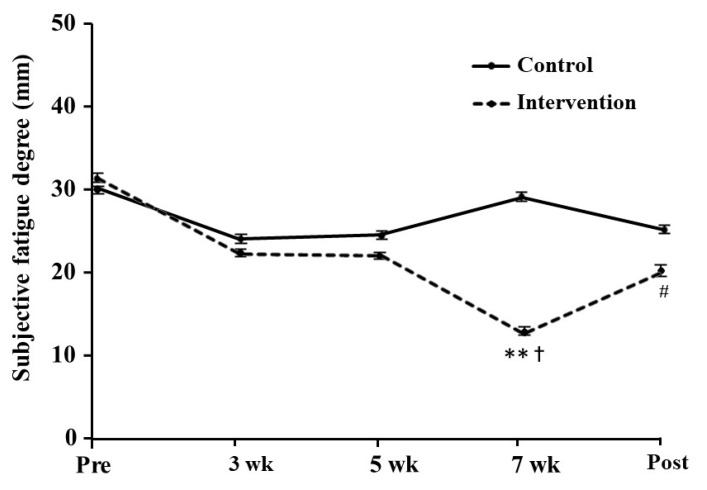
The degree of subjective fatigue at rest during the training period. The values are presented as mean ± SE. ** Significantly difference at *p* < 0.01 compared with before training. † Significantly different at *p* < 0.05 compared with the control group. # Difference at *p* = 0.070 compared with before training. Pre: pre-training, Post: post-training.

The subjective fatigue score at rest in the intervention group was significantly decreased at 7 weeks as compared with before training, with a large effect size (*p* = 0.006, *d* = 0.97; Figure 3). The lower score was maintained during the post-training period (*p* = 0.070, *d* = 0.58). By contrast, we found no significant difference in the control group.

### 3.6. Nutrients Intake

The consumptions of astaxanthin, β-carotene, and resveratrol during the intervention were significantly increased in the intervention group (*p* < 0.05) and significantly higher than those in the control group (*p* < 0.05; Table 3). The intakes of total energy, fat, carbohydrate, and vitamins A and C were not significantly changed between the groups, whereas the protein intake was significantly increased in the control group only (*p* = 0.023).

## 4. Discussion

Previous studies have investigated the effect of large amounts of micronutrients as supplement forms on health promotion and training-induced adaptation. However, most of these studies reported ineffective or detrimental rather than beneficial effects in humans. While increased consumption of fruit and vegetables containing micronutrients is recommended for health promotion [12], the effect of dietary intervention on training-induced adaptation remains unclear. In this study, we found that the increase in MVC in response to 10-week training was larger in the participants with intakes of dietary astaxanthin-, β-carotene-, and resveratrol-rich foods. In addition, resting oxygen consumption was increased by training in the intervention group only. These results suggest that dietary intervention with astaxanthin-, β-carotene-, and resveratrol-rich foods supported resistance training-induced adaptation.

Skeletal muscle mass was increased by resistance training in both the control and intervention groups. In general, the hypertrophy of individual fibers is caused by activated protein anabolism. Previous studies have suggested that astaxanthin, β-carotene, and resveratrol activate the mTOR/p70S6K protein synthesis signal in muscle tissues and cultured myotubes [7,8,9]. Recently, we demonstrated that the combined intake of these three factors, even in small amounts, promotes muscle hypertrophy during the hypertrophic period along with activation of the mTOR/p70S6K protein synthesis signal [10]. This effect suggests that the combined intake of these micronutrients could be more beneficial to the process of muscle adaptation than a higher intake of any single factor [13]. Strength is well known to increase adaptively in the early training period, and hypertrophy is subsequently observed with an extended training period. In the present study, however, no significant difference was found between the groups. Considering the higher MVC in the intervention group, the increase in muscle mass and the different types of protein in the areas closely related to muscle exertion may have an effect. Indeed, recent studies have shown that hypertrophy is more evident at the origin and insertion of the muscle [14]. Further studies are required to confirm the hypertrophic effects in longer intervention studies. On the other hand, increased intake of dietary protein during resistance training enhances muscle protein synthesis [15]. We found that dietary protein intake during the training period was increased in the control group only, which might have positively affected muscle hypertrophy. Nevertheless, an equal increase in muscle mass in both groups indicated no detrimental effect of the intervention. In contrast to muscle mass, muscle strength was increased in the intervention group only. This suggested an association of other factors, but not muscle hypertrophy.

Activity of the motor units, such as recruitment and frequency of transmission of motor neurons and neuromuscular synapses, are possible factors. Some food factors can promote neuromuscular efficiency and increase peak force production [16,17]. Other factors can also stimulate muscle fibers to participate in contraction by activating sympathetic nerves [18], which leads to an improved MVC. In addition, actomyosin formation, which is the interaction between actin and myosin, contributes to muscle strength. Antioxidant treatment improves the stabilization of neuromuscular synapses, which leads to the improvement of actomyosin interaction and force generation [19]. The present study examined the effect of the initial training period that brought about strength improvement by fiber mobilization to contraction, not muscle hypertrophy. Therefore, the nutritional factors astaxanthin, β-carotene, and resveratrol might stimulate neuromuscular interaction and actomyosin formation, resulting in the improvement of muscle strength.

Another possible factor for strength improvement is the regulation of cytosolic calcium homeostasis. Intracellular calcium concentration is regulated by calcium release from and uptake into the endoplasmic reticulum in muscle cells. When the action potential is propagated to the muscle sheath, calcium is released from the sarcoplasmic reticulum into the muscle cytoplasm through a calcium channel, ryanodine receptor [20]. The elevated calcium concentration causes muscle contraction through actin–myosin interaction. As the ryanodine receptor is post-translationally modified by reactive oxygen species (ROS), excessive oxidation can prevent calcium release and weaken muscle contraction [21]. Astaxanthin, β-carotene, and resveratrol might prevent oxidative modification of the receptor through their antioxidative properties [22,23].

Astaxanthin and resveratrol have been shown to promote aerobic metabolism in the skeletal muscle. Resveratrol supplementation for 4 weeks accelerated training-induced mitochondrial activation in the skeletal muscle of humans [24]. In animal studies, 15 weeks of resveratrol supplementation improved oxygen consumption in muscle cells [25]. Astaxanthin supplementation also increased mitochondrial biogenesis [26]. In the present study, oxygen consumption at rest increased after resistance training in the intervention group, which suggests elevated energy expenditure via activation of the mitochondria. The effects of resistance training also include improvements in glucose uptake and aerobic metabolic capacity. The increase in oxygen uptake in the intervention group suggests that the three components may have favored this metabolic adaptation as well [27,28]. In addition, dietary intervention could also affect substrate utilization. Resistance training adaptively decreases the respiratory quotient at rest [29], as observed in the control group of this study. However, it did not change after training in the intervention group. Previously, we showed that intervention with astaxanthin- and polyphenol-rich foods, combined with training, promoted carbohydrate oxidation at rest [30]. Such an effect might lead to unchanged substrate utilization, and astaxanthin and resveratrol potentiated the metabolism of mitochondria in muscle cells.

Energy in the skeletal muscle is mainly produced aerobically in mitochondria. In the process of aerobic metabolism, ROS is generated with oxygen consumption during exercise [31]. Excessive ROS levels attenuate muscle strength and cause cell inflammation [32,33]. By contrast, moderate oxidative stress can mediate the expression of internal antioxidants and the activation of mitochondria [33]. Therefore, preventing exercise-induced ROS does not always lead to improved skeletal muscle function. Indeed, many studies have reported the negative effects of high-dose antioxidants on exercise performance, which inhibit the beneficial effects of exercise, such as improvement of energy metabolism, strength, and endogenous antioxidant activity [34,35,36]. Focusing on muscle adaptation, several studies showed that high doses of vitamins C and E either attenuate or do not affect muscle adaptation by resistance training in humans [36,37]. By contrast, β-carotene and resveratrol have been reported to enhance protein synthesis signaling, including Akt, mTOR, and p70S6K, in skeletal muscle tissues and cultured myotubes [7,8,9]. Furthermore, we previously showed that the intake of astaxanthin, β-carotene, and resveratrol promoted muscle hypertrophy with activated p70S6K signaling in mice [10]. In the present study, the intervention did not attenuate muscle adaptation but rather enhanced strength and energy expenditure without affecting muscle mass.

Serum carbonylated protein levels immediately after exercise in the post-training period tended to be decreased in the intervention group, which suggests an antioxidant effect of the dietary intervention. Carbonylated protein is a sensitive biomarker of oxidative damage elevated by exercise intervention, which is detected as an amino acid residue that reacts with lipid peroxide. Exercise-induced ROS and free radicals are important signaling factors in physiological responses and adaptations and also act as pro-inflammatory factors [38]. This effect depends on the intensity and duration of exercise, exercise style, diet, and individual physical characteristics. When the oxidative stress caused by the balance between the generated ROS and the antioxidant capacity is at a moderate level, it tends toward the former, but when at a high level, it tends toward the latter. Excessive oxidative stress can be involved in muscle fatigue and damage induced by high-intensity exercise [39]. In addition, we found that training-induced fatigue was lower in the intervention group. Previously, dietary polyphenol-rich foods suppressed muscle damage after exercise [40,41]. Other studies have shown that mitochondrial ROS production may contribute to neuromuscular junction instability [42,43]. Carnio et al. [19] also suggested that mitochondrial dysfunction and ROS directly prevent actomyosin interaction and force generation. Astaxanthin and β-carotene are lipid-soluble carotenoids present in cell membranes [44], while resveratrol is a water-soluble polyphenol that exists in cell structures outside the membrane, such as the cytoplasm, granules, and vacuoles [45]. Therefore, these different characteristics might interact when they are taken together even at a small dose. Indeed, several antioxidants effectively work when consumed in combination [46,47]. The combined intake of antioxidants from foods may have some effects on the reduction of ROS and promotion of muscle adaptation.

A limitation of this dietary intervention study is that it was difficult to clarify whether the benefits were obtained by a specific ingredient or the combined effect of the whole. In addition, the basal diet and intake of nutrients were difficult to unify among participants, as a higher intake of vitamin E was observed in the intervention group. In contrast to other forms of supplements, the daily intake of each nutrient from foods was lower. However, the combined intake of multiple ingredients might exert the benefits without the negative effects of excess antioxidants. Furthermore, many other ingredients in addition to targeted micronutrients can be taken, which may also support the benefit. Nevertheless, only a few studies have investigated the effect of functional nutrient-rich foods on training-induced adaptation in humans. Although body composition was measured with BIA in this study, accurate changes in skeletal muscle mass could be clarified by using dual-energy X-ray absorptiometry or magnetic resonance imaging (MRI). Further research is required to clarify muscle adaptation over longer intervention periods and in participants with different characteristics, such as athletes and elderly people, in the future.

In conclusion, this study showed that a daily intake of astaxanthin-, β-carotene-, and resveratrol-rich foods enhanced the adaptation of muscle strength and resting energy metabolism induced by 10 weeks of resistance training. Subjective fatigue and oxidative damage were moderated by the intervention, which might support training-induced adaptation. The anabolic micronutrient-rich foods may increase training efficiency in the early period of resistance training.

## Figures and Tables

**Table 1:** Physical characteristics of subjects in pre- and post-resistance exercise training.

Physical Characteristics	Control			Intervention		
	Pre	Post	Change	Pre	Post	Change
Body weight (kg)	59.7 ± 1.9	60.0 ± 1.9	0.10 ± 0.60	59.1 ± 1.6	59.7 ± 1.5	0.56 ± 0.62
BMI (kg/m^2^)	20.4 ± 0.5	20.6 ± 0.5	0.24 ± 0.23	20.3 ± 0.5	20.4 ± 0.5	0.10 ± 0.10
Body fat (%)	14.3 ± 1.4	13.9 ± 1.3	−0.44 ± 0.37	14.8 ± 1.2	14.6 ± 0.9	−0.18 ± 0.45
Skeletal muscle mass (kg)	28.4 ± 0.8	28.8 ± 0.8 *	0.48 ± 0.14	28.3 ± 0.7	28.6 ± 0.7 *	0.31 ± 0.13
MVC of right leg (kg)	25.3 ± 1.9	26.8 ± 2.0	1.6 ± 0.8	26.0 ± 2.6	32.4 ± 2.5 **	6.4 ± 2.2
MVC of left leg (kg)	23.2 ± 2.3	25.6 ± 1.5	2.4 ± 1.2	24.7 ± 2.2	30.0 ± 2.2 **	5.0 ± 1.2

Mean ± SE, *n* = 13. * *p* < 0.05 vs. Pre, ** *p* < 0.01 vs. Pre. Pre; Pre-training, Post; Post-training. BMI; body mass index, MVC; maximal voluntary contraction.

**Table 2:** Comparison of metabolic performance in pre- and post-resistance exercise training.

Metabolic Parameters	Control			Intervention		
	Pre	Post	Change	Pre	Post	Change
Oxygen consumption (mL/kg/min)	3.42 ± 0.11	3.44 ± 0.08	0.02 ± 0.11	3.35 ± 0.10	3.57 ± 0.10 *	0.18 ± 0.10
Respiratory quotient	0.87 ± 0.01	0.83 ± 0.01 *	−0.03 ± 0.01	0.88 ± 0.01	0.86 ± 0.02	−0.02 ± 0.02
Carbohydrate utilization (mg/gB.W./min)	2.53 ± 0.19	2.05 ± 0.21 *	−0.49 ± 0.19	2.67 ± 0.16	2.57 ± 0.30	−0.10 ± 0.30
Fat utilization (mg/gB.W./min)	0.73 ± 0.05	0.94 ± 0.08 *	0.21 ± 0.08	0.66 ± 0.05	0.80 ± 0.09	0.13 ± 0.07

Mean ± SE, *n* = 13. * *p* < 0.05 vs. Pre. Pre; Pre-training, Post; Post-training.

**Table 3:** Daily intake of nutrients and antioxidants in pre and 10 weeks resistance exercise training.

Energy and Nutrients	Control		Intervention	
	Pre	10 Weeks	Pre	10 Weeks
Energy (kcal/d)	2144 ± 92	2352 ± 112	2150 ± 91	2371 ± 105
Protein (g/d)	80.0 ± 5.2	93.6 ± 4.2 *	95.5 ± 5.2	107.8 ± 6.1
Fat (g/d)	70.4 ± 5.7	77.0 ± 7.3	71.8 ± 5.4	74.2 ± 5.8
Carbohydrate (g/d)	283.0 ± 16.7	306.0 ± 20.0	302.8 ± 15.7	310.7 ± 18.2
Vitamin A (µg/d)	423 ± 31	416 ± 56	833 ± 309	824 ± 272
Vitamin C (mg/d)	51 ± 5	70 ± 9	63 ± 3	91 ± 11
Vitamin E (mg/d)	7 ± 0.6	8 ± 0.6	7 ± 0.5	11 ± 0.7 ^†^
Astaxanthin (mg/d)	0.23 ± 0.08	0.21 ± 0.08	0.3 ± 0.12	1.87 ± 0.48 ** ^†^
β-carotene (µg/d)	2141 ± 235	2048 ± 209	2126 ± 175.5	12,130 ± 609.6 ** ^†^
Resveratrol (µg/d)	0	0	0	45 ** ^†^
Salt (g/d)	9.0 ± 0.4	10.0 ± 0.6	10.0 ± 0.6	9.5 ± 0.7

Mean ± SE, *n* = 13. * *p* < 0.05 vs. Pre, ** *p* < 0.01 vs. Pre, ^†^ *p* < 0.01 vs. Control. Pre; Pre-training.

## Data Availability

The data presented in this study are available on request from the corresponding author. The data are not publicly available.
